# Primary hemiarthroplasty after unstable trochanteric fracture in elderly patients: mortality, readmission and reoperation

**DOI:** 10.1186/s12891-021-04277-7

**Published:** 2021-04-30

**Authors:** Tzu-Chieh Lin, Pin-Wen Wang, Chun-Teng Lin, Yu-Jun Chang, Ying-Ju Lin, Wen-Miin Liang, Jeff Chien-Fu Lin

**Affiliations:** 1 Department of Public Health, China Medical University, Taichung, Taiwan; 2 Department of Emergency Medicine, Taichung Veterans General Hospital, Taichung, Taiwan; 3 Department of Health Services Administration, China Medical University, No. 100, Sec. 1, Jingmao Rd, Taichung, 406040 Taiwan; 4 Big Data Center, Epidemiology and Biostatistics Center, Changhua Christian Hospital, Changhua, Taiwan; 5 Genetic Center, Proteomics Core Laboratory, Department of Medical Research, China Medical University Hospital, Taichung, Taiwan; 6 School of Chinese Medicine, China Medical University, Taichung, Taiwan; 7 Department of Statistics, National Taipei University, No.67, Sec. 3, Ming-Shen E. Rd, Taipei 10478 Taipei, Taiwan; 8 Department of Orthopedic Surgery, Wan Fang Hospital, Taipei Medical University, Taipei, Taiwan

**Keywords:** Unstable trochanteric fracture, Hemiarthroplasty, Mortality, Readmission, Reoperation

## Abstract

**Background:**

Most unstable trochanteric fractures are treated with internal fixation and often with high complication rates. Hemiarthroplasty might be an alternative method in difficult condition, especially in unstable comminuted fracture in fragile bone. However, few have investigated the long-term outcomes after hemiarthroplasty for unstable trochanteric fracture. We conducted a population-based retrospective cohort study of trochanteric fracture after primary hemiarthroplasty using competing risk analysis on their long-term outcomes, including mortality, readmission and reoperation.

**Methods:**

We studied a total of 2798 patients over 60 years old, with a mean age of 79 years, of which 68% are females and 67.23% have at least one comorbidity. They underwent a hemiarthroplasty for unstable trochanteric fracture during the period between January 1, 2000 and December 31, 2010 and were follow-up until the end of 2012, or death. Survival analysis and Cox model were used to characterize mortality. Competing risk analysis and Fine and Gray model were used to estimate the cumulative incidences of the first readmission and the first reoperation.

**Results:**

The follow-up mortality rate for 1-year was 17.94%; 2-year, 29.76%; 5-year, 56.8%; and 10-year, 83.38%. The cumulative incidence of the first readmission was 16.4% for 1-year and 22.44% for 3-year. The cumulative incidence of the first reoperation was 13.87% for 1-year, 18.11% for 2-year, 25.79% for 5-year, and 38.24% for 10-year. Male gender, older age, higher Charlson Comorbidity Index (CCI) and lower insured amount were all risk factors for the overall mortality. Older age and higher CCI were risk factors for the first readmission. Older age was a protective factor for reoperation, which is likely due to the competing death.

**Conclusions:**

The mortality and revision rates after hemiarthroplasty for unstable trochanteric fracture are acceptable as a salvage procedure for this fragile sub-population.

**Supplementary Information:**

The online version contains supplementary material available at 10.1186/s12891-021-04277-7.

## Background

The incidence of osteoporotic hip fracture is increasing along with the expanding elderly population. Its treatment could end with excess mortality, morbidity and socioeconomic burden [1–3]. Osteoporotic hip fractures include either cervical fracture or the trochanteric fracture of femur is about 1:1 ratio of incidence. Unstable trochanteric fracture is defined as a comminution of posteromedial cortex, reverse obliquity and subtrochanteric extension. Classifications of trochanteric fractures are not useful for selecting the optimal treatment. The optimal choice of operation and implant for unstable trochanteric fracture remains debatable [4, 5]. Most unstable intertrochanteric fractures are treated with internal fixation. However, internal fixation often leads to multiple surgical complications, like loss of fixation, lag screw cut-out, collapse, malunion and implant failure. All these result in reoperation or switching to arthroplasty [6, 7]. Therefore, hemiarthroplasty might be an alternative treatment for unstable trochanteric fracture, such that bone quality is poor or a multi-fragmentary or comminuted fracture occurred [7–10]. Grau et al. reported that 3.3% of 2,117,815 intertrochanteric fractures were treated by hemiarthroplasty and the use of hemiarthroplasty to treat trochanteric fracture has increased over the period from 1990 to 2007 [7].

Recently, a number of studies, typically enrolled limited samples, have reported the short-term outcomes after hemiarthroplasty to treat unstable trochanteric fractures [8–18]. Furthermore, few studies investigated the long-term outcomes [11, 15–17]. Here, we conducted a population-based retrospective cohort study on trochanteric fracture investigating the mid- and long-term outcomes (like overall mortality, readmission and reoperation) after primary hemiarthroplasty. The method of competing risk analysis was used.

## Methods

### Data sources

Data were retrospectively collected from two sources: the National Health Insurance Research Database (NHIRD) (details available at: http://nhird.nhri.org.tw/en/index.htm) and the National Register of Deaths Database (NRDD). These databases and another 70 health-related databases are real-world data and maintained by Taiwan’s Ministry of Health and Welfare (MOHW) Data Center. The completeness and accuracy of these two databases are > 98% and are endorsed by MOHW. Taiwan National Health Insurance (NHI) program was started in 1995, providing mandatory health insurance for ≥99% of all the 23 million residents in Taiwan. The NHIRD contains individuals’ e-claims of all NHI beneficiaries from 1996 to the present. NHIRD and other databases maintained by MOHW Data Center are real-world data, and providing real-world evidence for healthcare policy making. Our study was approved by the Research Ethics Committee, China Medical University Hospital (certificate: CMUH-104-REC2–115).

### Patients

Like our previous report [19], inclusion criteria were patients aged 60 years or older with the following two conditions: (a) a first discharge diagnosis code of trochanteric hip fracture based on the International Classification of Diseases, 9th Revision, Clinical Modification (ICD-9-CM) codes 820.20, 820.21, 820.30 and 820.31, and (b) a procedure code corresponding to surgery for hemiarthroplasty based on ICD-9-CM codes 81.52 during the period from January 1, 2000 to December 31, 2010. The first admission date for trochanteric fracture was defined as the index date of surgery. The exclusion criteria were patients with pathological fractures (ICD-9-CM codes 733.14 and 733.15) or open hip fractures (ICD-9-CM codes 820.1, 820.10, 820.11, 820.12, 820.19, and 820.9). To avoid confounding effects, we excluded patients undergoing surgery before the index date for injuries to the pelvis, femur, or hip region. We extracted a total 2798 patients receiving hemiarthroplasty for trochanteric fracture. They were among a total 76,798 patients, aged of 60 or older, admitted to hospitals with a primary diagnosis of trochanteric fracture during the study period. According to the guideline of the National Health Insurance (NHI) program in Taiwan, the relative indications of hemiarthroplasty for trochanteric fracture are patients aged 60 years or older with acute unstable trochanteric fractures which is defined as a comminution of posteromedial cortex, reverse obliquity and subtrochanteric extension (i.e., AO/ASIF types 31: A2.2, A2.3, A3.1, A3.2, and A3.3) based on Modified Jensen-Evans classification [20] and AO/ASIF classifications [21]. However, neither the classifications of Evans nor AO/ASIF classifications could definitely guide the indications for hemiarthroplasty. Consequently, all the hemiarthroplasty operations were reviewed through a peer-review system within NHI program, by at least three board-certificated orthopedic surgeons and they approved the proposed operations. The comorbidities of each patient were retrieved before or at the time of the index day on the basis of Charlson comorbidity index (CCI). The end of the study was either the date of death, the date of exiting from NHI program, or December 31, 2012, the last follow-up date.

### Outcomes of interest

Three endpoints were studied: (a) overall cumulative mortality, (b) cumulative incidence of major medical complication resulting prolonging hospitalization or rehospitalization, and (c) cumulative incidence of surgical complication resulting in unplanned reoperation. From the NHIRD database, we identified the first readmission due to medical complications with discharge diagnosis codes by ICD-9-CM after the index surgery. There is no clear cut-off point between medical complications and newly developed comorbidities after major surgery. We, therefore, interpreted a shorter duration from the index day to the day of the first medical readmission as indicating a greater probability that the readmissions were caused directly or indirectly by the surgery. We assume that medical complications requiring re-hospitalization that occur within 6 months after index surgery were considered as the postoperative medical complications. The 6-month medical complications included stroke, acute myocardial infarction, pulmonary embolism, acute renal failure, and acute respiratory failure. We also identified the first reoperation due to surgical complications based on the procedure codes and discharge diagnosis codes by ICD-9-CM after the index surgery. The causes of the reoperations included surgical site infection, conversion to total hip arthroplasty or revision arthroplasty, removal of prostheses, mechanical complications (loosening of stem, break of fixed steel wire/cable and prosthesis failure), dislocation and periprosthetic fracture at the same site during the follow-up period. The overall survival time was calculated from the index date to the date of death. The first readmission time was the time from the index date to the date of the first readmission due to medical complications that required hospital readmission, within 6 months after the index surgery. The first reoperation time was calculated from the index date to the date of the first postoperative unplanned reoperation due to surgical complications secondary to hemiarthroplasty. Patients alive at the end of the follow-up period or alive without any complication at the end of follow-up period were treated as censored.

### Statistical analysis

Overall survival rates were estimated using the Kaplan-Meier method. We explored effects of risk factors on the overall survival using both the log-rank test and multiple variables Cox’s proportional hazards model. Elderly patients in our study typically had a high mortality rate due to ageing and multiple comorbidities. A high competing risk of death could interfere with the estimation of cumulative incidences on the first readmission and the first reoperation. The Kaplan-Meier method overestimates the cumulative incidences of the first readmission and the first reoperation if not considering competing death. Therefore, we estimated these cumulative incidences using the competing risk analysis with death treated as a competing event. In addition, we also explored risk factors on the first readmission time and the first reoperation time using the Gray’s test and the Fine and Gray’s model. These methods are multivariate subdistribution hazard models based on competing risk analysis [22, 23]. All data management and analyses were performed using the statistical package SAS 9.4 (SAS Institute, Cary, NC) and R libraries survival, cmprsk, and mstate, R 3.6.1 (available at http://www.R-project.org/, R Development Core Team (2019), R: A language and environment for statistical computing. (R Foundation for Statistical Computing, Vienna, Austria).

## Results

During 2000 to 2010, a total of 76,798 patients aged 60 years or older were admitted for the first time with a primary diagnosis of trochanteric fracture. Of these, 2798 patients were included in the study. They satisfied the inclusion/exclusion criteria and underwent hemiarthroplasty as the primary treatment for trochanteric fracture. As shown in Table 1, the cohort consisted of 1903 (68.0%) women and 895 (32%) men. Their mean age was 79 years old. Females had slightly higher mean age, lower CCI score and higher insurance amount.

**Table 1:** Baseline characteristics of patients receiving hemiarthroplasty for intertrochanteric hip fractures stratified by gender

				Gender				*P*-value
		Total		Male		Female		
		(*n* = 2798)		(*n* = 895)		(*n* = 1903)		
		N	%	N	%	N	%	
Age (yrs)	Mean ± SD^a^	79.75 ± 7.86		78.74 ± 7.78		80.23 ± 7.85		< 0.001
	60–64	113	4.04	48	5.36	65	3.42	
	65–69	226	8.08	82	9.16	144	7.57	
	70–74	407	14.55	139	15.53	268	14.08	
	75–79	599	21.41	209	23.35	390	20.33	
	80–84	737	26.34	236	26.37	501	26.33	
	≥ 85	716	25.59	181	20.22	535	28.11	
CCI^b^ score	0	917	32.77	256	28.60	661	34.73	< 0.001
	1	694	24.80	203	22.68	491	25.80	
	2	372	13.30	137	15.31	235	12.35	
	3	228	10.29	102	11.40	186	9.77	
	≥4	527	18.83	197	22.01	330	17.34	
Insurance amount (NT$)	≤21,000	1704	60.90	640	71.51	1064	55.91	< 0.001
	> 21,000	1094	30.10	255	28.49	839	44.09	

*Note*: ^a^ *SD* Standard deviation

^b^ *CCI* Charlson Comorbidity Index

**Fig. 1:**
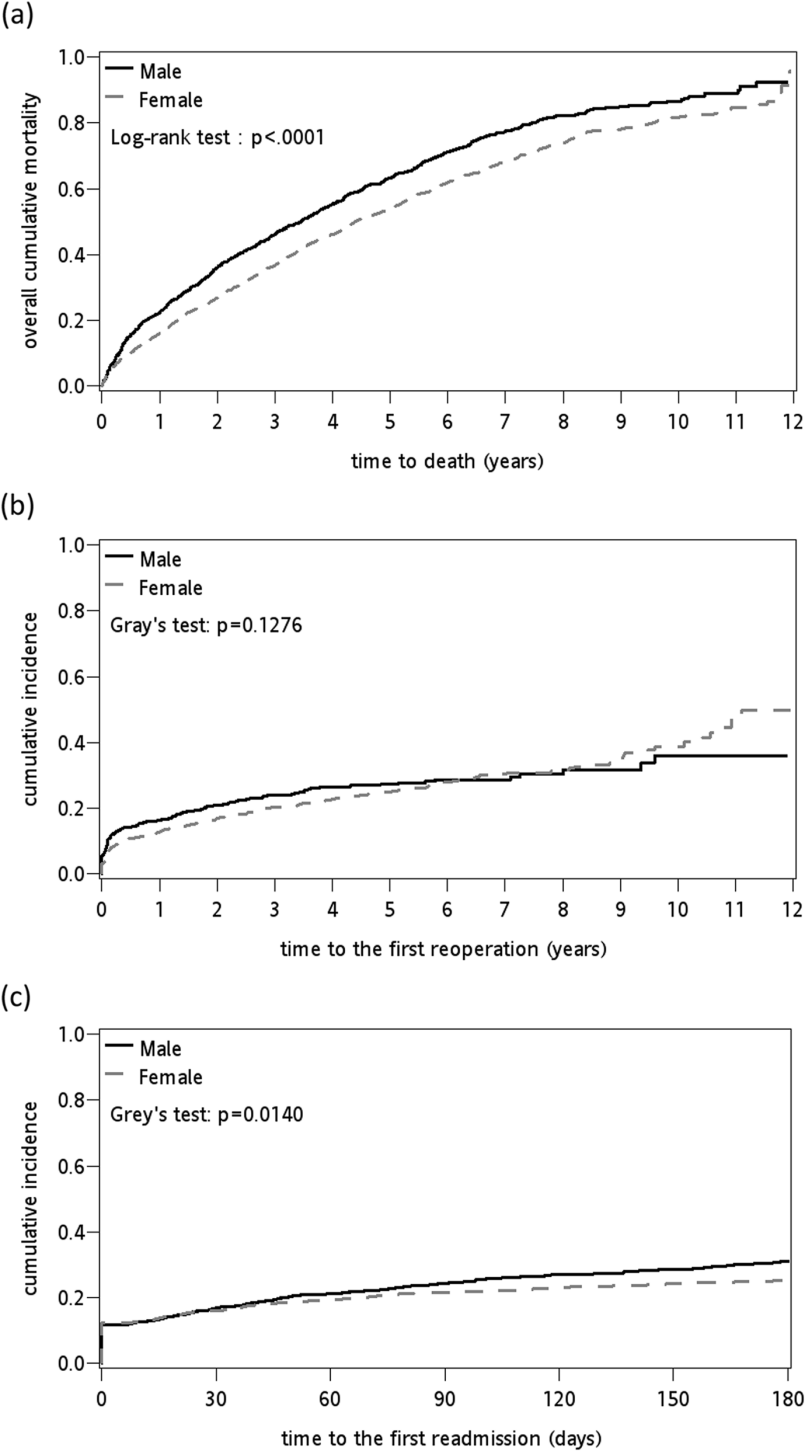
Twelve-year cumulative incidence curves of (**a**) mortality stratified by gender, (**b**) the first reoperation stratified by gender, and (**c**) 180-day cumulative incidence curves of the first readmission stratified by gender

The 1-month, 3-month, 6-month, 1-year, 2-year, 5-year and 10-year cumulative mortality rates were 3.15, 5.5, 7.4, 17.94, 29.76, 56.8 and 83.38%, respectively (Table 2). The 1-, 2-, 5-, and 10-years cumulative incidences of the first reoperation for surgical complications were 13.87, 18.11, 25.79 and 38.24%, respectively. The 1-, 3-, and 6-month cumulative incidences of the first readmission for medical complications were 16.4, 22.44 and 27.13%, respectively. Females had lower cumulative mortality after 12-year follow-up (Table 2 and Fig. 1 a). Females had lower cumulative incidence of the first reoperation before 5-year follow-up; then, slightly higher cumulative incidences of the first reoperation after 8-year follow-up (Table 2 and Fig. 1 b). Females had lower cumulative incidence of the first readmission (Table 2 and Fig. 1 c).

**Table 2:** Cumulative mortality rates and cumulative incidence of first reoperation or readmission after hemiarthroplasty for intertrochanteric hip fractures

				Cumulative Incidence					
	Cumulative mortality (%)			Reoperation (%)			Readmission (%)		
Time	Total	Male	Female	Total	Male	Female	Total	Male	Female
1-month	3.15	3.24	3.10	6.55	8.43	5.67	16.40	16.98	16.13
3-month	5.50	6.48	5.04	10.06	12.77	8.79	22.44	24.58	21.65
6-month	7.40	8.83	6.73	11.93	14.38	10.83	27.13	31.06	25.28
1-year	17.94	22.23	15.92	13.87	16.38	12.81			
2-year	29.76	35.93	26.84	18.11	21.02	16.90			
5-year	56.80	63.11	53.78	25.79	27.69	25.20			
10-year	83.35	86.65	81.79	38.24	36.00	40.14

The statistically significant risk factors for the overall survival were male gender, older age, higher CCI score and lower insured amount (Table 3). For each 1-year increase in age, the hazard ratio (HR) increased by 1.056 (95% CI: 1.047–1.064). Males had death risks higher than females, HR, 1.315 (95% CI: 1.166–1.482). Patients with higher CCI scores had higher risks for death compared with those with CCI score equal to 0 (CCI = 1, HR 1.14, 95% CI: 1.12–1.55; CCI = 2, HR 1.69, 95% CI: 1.40–2.04; CCI = 3, HR 1.77, 95% CI: 1.45–2.16; CCI ≥ 4, HR 2.43, 95% CI: 2.07–2.86, respectively). The significant risk factor for the first reoperation was only younger age. For each 1-year increase in age, the sub-distribution hazard ratio (sHR) decreased 1.4% (sHR: 0.986, 95% CI: 0.974–0.998) (Table 3). Conversely, the significant risk factors for the first readmission were older age and higher CCI score. For each 1-year increase in age, the sub-distribution hazard ratio (sHR) increased by 1.024 (95% CI: 1.016–1.032).

**Table 3:** (a) Hazard ratios of the risk factors associated with death from cause-specific hazard model based on multivariate Cox’s model, (b) subdistribution hazard ratio of the risk factors associated with the first reoperation, and (c) first readmission based on multivariate Fine and Gray’s (subdistribution hazard ratio, sHR) model from competing risk analysis

		(a) Death			(b) Reoperation			(c) Readmission		
		HR	95% C.I.	*P*-value	sHR	95% C.I.	*P*-value	sHR	95% C.I.	*P*-value
Age (year)		1.056	(1.047–1.064)	< 0.001	0.986	(0.974–0.998)	0.025	1.024	(1.016–1.032)	< 0.001
Gender	Female	Ref.^b^			Ref.			Ref.		
	Male	1.315	(1.166–1.482)	< 0.001	0.966	(0.770–1.213)	0.768	0.951	(0.829–1.090)	0.468
CCI^a^	0	Ref.			Ref.			Ref.		
	1	1.139	(1.123–1.548)	< 0.001	1.038	(0.797–1.351)	0.784	1.365	(1.172–1.590)	< 0.001
	2	1.693	(1.404–2.041)	< 0.001	0.898	(0.634–1.270)	0.542	1.684	(1.388–2.044)	< 0.001
	3	1.766	(1.445–2.159)	< 0.001	1.087	(0.764–1.547)	0.642	1.656	(1.320–2.077)	< 0.001
	≥ 4	2.432	(2.070–2.856)	< 0.001	0.886	(0.648–1.210)	0.447	2.103	(1.752–2.524)	< 0.001
Insurance amount (NT$)	≤ 21,000	Ref.			Ref.			Ref.		
	> 21,000	0.487	(0.425–0.557)	< 0.001	1.174	(0.950–1.450)	0.138	0.912	(0.803–1.036)	0.158

*Note*: ^a^ *CCI* Charlson Comorbidity Index

^b^ *Ref*. Reference

Patients surviving after index surgery typically had multiple reoperations and readmissions due to complications. A total of 356 patients (12.72%) had at least one reoperation within 10 years after index surgery (Table 4). Causes for reoperation were dislocation (30.4%), infection (40.73%), periprosthetic fracture (20.5%) and mechanical complications (41.2%). Surgical complications resulted in 40 revisions, 11.8% among 356 patients after the first reoperations and 1.4% among total 2798 patients after the index surgery. For patients receiving reoperation after index surgery, the cumulative mortality rates after the first reoperation within 1-month, 3-month, 6-month, 1-year, 2-year, 5-year follow-up were 4.5, 13.67, 19.45, 28.62, 39.25 and 61.95%, respectively. A total of 759 patients (27.13%) had at least one readmission within 6 months after the index surgery (Table 4). Causes for readmission in descending order were urinary tract infection (60%), pneumonia (37%), acute respiratory failure (25%), acute renal failure (10.9%) and major cardiovascular events. Cumulative mortality rates after first medical readmission within 1, 3 and 6 months after the index surgery were 14.17, 23.61 and 30.85%, respectively.

**Table 4:** Causes of the first reoperation or readmission after hemiarthroplasty for intertrochanteric hip fracture

	1-month	3-month	6-month	1-year	2-year	5-year	10-year
Reoperation							
N^a^	127	199	228	250	283	330	356
Dislocation (%^b^)	36.22	41.71	40.97	40.00	36.04	31.82	30.34
Mechanical complications^c^ (%)	35.43	36.18	37.72	39.20	40.99	41.21	40.43
Infection (%)	34.65	35.18	39.04	39.60	36.58	41.21	40.73
Removal of implant (%)	14.17	13.07	14.91	16.00	18.37	20.30	20.79
Conversion to / revision arthroplasty (%)	4.72	6.03	6.14	7.60	10.60	11.82	11.80
Isilateral femur fracture (%)	14.17	15.08	14.47	14.80	17.67	19.09	20.51
Readmission							
N	459	628	759				
Urinary tract infection (%)	55.34	58.28	60.87				
Pneumonia (%)	28.54	33.28	37.42				
Acute respiratory failure (%)	17.86	22.29	25.30				
Stroke (%)	10.46	11.15	10.67				
Acute renal failure (%)	8.50	11.31	10.94				
Acute myocardial infarction (%)	4.14	4.62	4.48				
Pulmonary embolism (%)	1.96	1.59	1.71				
Deep vein thrombosis (%)	0.87	1.43	1.45				

*Note*: ^a^ *N* = the number of subjects who had at least one reoperation or readmission

^b^ % = percentage of subjects who had a certain cause of complication among the total number of subjects who had at least one complication. Subjects might have had more than one reoperation or readmission due to multiple causes

^c^ Mechanical complications include loss of reduction, cortex penetration by stem, skin irritation and implant broken/failure

## Discussion

Several other studies reported the short-term outcomes after hemiarthroplasty for unstable trochanteric fracture with various results [8–18]. Their sample sizes are rather small (from 29 to 277) and most (> 70%) of them had sample sizes < 100 [8–10, 12–14, 18, 24–30]. Discrepancy in mortality rates among studies could be due to differences in selection criteria, distributions of gender and age in the populations, pre-fracture physical activity, bone quality, nutrition, and comorbidities, in addition to sample sizes. Our patients’ one-month mortality was 3.15%, which is lower than those reported in the literature (i.e., 4 to 13.8%) [9–12, 14, 30]. One possible reason is that our NHI healthcare program provides all essential surgical and medical treatments free to patients during hospital admissions. The 3-month mortality of our patients was 5.5%, which is lower than those reported in the literature (i.e., 7.0 to 26.6%) [10, 12, 30]. Our 6-month mortality was 7.42%, which is also lower than those reported in the literature (i.e., 23.5 to 26.4%) [10, 24, 29]. Cornwall et al. investigated the short-term mortality rates of 4 types of hip fractures [31]. Their in-hospital and 6-month mortality rates were 0 and 5.7% for 70 non-displaced femoral neck fractures, 2.2 and 15.8% for 181 displaced femoral neck fractures, 2.8 and 12.8% for 108 stable intertrochanteric fractures and 1.1 and 13.8% for 178 unstable intertrochanteric fractures [31]. When compared with the previous studies, mortality rate of our patients increased more rapidly after 6-month follow-up. Our patients’ 1-year mortality was 17.9%, which is higher than those reported in the previous studies (i.e., 2.5 to 14.6%) [13, 27, 28], but our 1-year mortality was still lower than 21.8 to 39.3% in most past studies [9–12, 14–18, 30]. Our 2-year mortality rate was 29.7%, which falls in the mid-range of 12.5 to 59.0%, as reported in the literature [8, 9, 12, 16, 27, 30]. Our 5-year mortality rate was 56.8% which was slightly higher than 52 to 64%, as reported in the literature for all hip fractures [2, 3, 19, 32]. Few studies reported 5-year mortality rates after hemiarthroplasty for unstable intertrochanteric fracture [16, 17]. Camurcu et al. and Cobden et al. reported their 5-year mortality rates as high as 94.4 and 90.25%, respectively [16, 17]. One important reason for the large differences in mortality rates between studies of ours and others [16, 17] is related to their smaller sample sizes. For a study with a smaller sample size study containing high mortality elderly patients, only a few number of survival patients were left toward the end of the study such that a small number of deaths would cause huge impact on the change of the mortality rate and cause a sharp rise for the mortality rate at the end of the study.

Our short-term mortality rates after hemiarthroplasty for unstable trochanteric fracture are not higher than those after hemiarthroplasty reported for cervical fracture and internal fixation for trochanteric fracture. For example, Forte et al. found 1-, 2-, and 3-month mortality rates among 192,365 elderly after internal fixations for trochanteric fractures are 7.92, 12.34 and 15.19%, respectively [33]. The meta analyses of Mundi and Li et al. for the outcomes after internal fixation for trochanteric fractures found the 1-year mortality rate being 23% after year 2000 and 17% [34, 35]. Tucker et al. conducted a prospective study including 3230 unstable trochanteric fractures with internal fixations and found the 1-year mortality rate being 22.6% [36]. Mattisson et al. reported a study for trochanteric fracture based on a database from Swedish fracture register and found that the overall 30-day and 1-year mortality rates being 7.7 and 26% [37]. In contrast, our 5-year and 10-year mortality rates after hemiarthroplasty for unstable trochanteric fracture were 56.8 and 83.3%, which are all higher than those reported in the literature after hemiarthroplasty for cervical fracture [2, 3, 19, 32]. Lin and Liang examined the outcomes of patients after hemiarthroplasty for displaced cervical fracture. They reported the 5-and 10-year mortality rates being 46.9 and 71% [19]. Studies reported that patients with trochanteric fractures tended to be older, in worse health conditions and higher short-term mortality rates than those with femoral neck fractures [29, 38]. We believe that unstable trochanteric fracture with sequelae and aging could result in high mortality as found in 1 year after fractures.

Other main findings of our study are the significant risk factors for overall survival rate being male gender, older age, higher CCI score and lower insured amount. Few studies reported on the risk factors for hemiarthroplasty after unstable trochanteric fracture [17]. Camurcu et al. reported 106 patients after cementer bipolar hemiarthroplasty for unstable trochanter fracture and found that risk factors for 1-year mortality being American Society of Anesthesiologists (ASA) scores ≧ 3, delayed postoperative mobilization ≧ 2 days and presence of ≧ 3 comorbidities. Camurcu et al. did not find age and male being risk factors for 1-year mortality. Several meta-analyses reported that older age, male gender and multiple preoperative comorbidities are significant risks for mortality and medical complications after hip fractures in the elderly [1, 39]. We found that males had a hazard 1.31 times of females and age had hazard yearly 1.05 times higher for the overall mortality. We used CCI score representing the severity of comorbidities. Other investigators used instead ASA score for unstable trochanteric fractures [11, 12, 14, 16, 25, 26, 30]. Higher CCI, aging, higher ASA scores and delayed surgery are highly correlated with one another. Multiple comorbidities and aging often result in high ASA scores. In such cases, longer waiting time is required to stabilize the pre-existing medical problems. We found that CCI score had stronger association with mortality than ASA score (data not shown here). CCI score or ASA score are in positive and strong correlation. They are both good measures for the number and severity of comorbidities. No consensus exists as to which measure is the best to represent the multiple comorbidities and how the measure should be integrated into the statistical analysis. Several studies had shown CCI score as a significant risk factor associated with the mortality after hip fractures [40, 41]. For this reason, we had chosen CCI score as the measure for the severity of multiple comorbidities.

Several other studies reported readmission rates and reoperation rates after hemiarthroplasty for unstable trochanteric fracture [9–11, 13–15, 18, 24, 27, 30]. However these studies did not consider the interferences caused by the competing risk of deaths in estimating the cumulative incidence of the readmission and the reoperation rates. It is therefore difficult to compare their findings with ours. Other difficulties are the large variations in sample size across studies, the differences in the definition of causes for the readmissions, the follow-up times and the lost to follow-up rates. Previous studies reported the 1- to 6-month rates of medical complications ranged from 11.2 to 41.8% for patients after hemiarthroplasty for unstable trochanteric fracture [9–11, 13, 18, 24, 27]. Several other studies reported the one-month readmission rates due to medical complications from 5.3 to 17.1% for all types of hip fractures [42–44]. Our cumulative incidences of the first readmission due to medical complications after hemiarthroplasty for trochanteric fracture seem higher than those cervical fracture reported in literature [9–11, 13, 18, 19, 24, 27, 42]. Patients with trochanteric fracture are older than those with cervical fracture of femur. Therefore, the first readmission rates are often higher in trochanteric fracture. We found that older age and higher CCI score are risk factors for the first readmission. In the literature review of Ali and Gobbons, they summarized that age, preoperative comorbidities are strong independent predictors of readmission after hip fracture operations [45]. Male gender, unlike for mortality, was not found to be a risk factor for readmission in our study. Pollock et al. did not find male gender being a risk for readmission in 1486 patients after hip fracture operations [46]. Lizaur-Utrilla et al. found that female gender, higher ASA score and more than 2 comorbidities are risk factors for readmission among 732 patients after hip fractures [47]. And French et al. also found that female gender and multiple comorbidities are risk factors for readmission in 41,331 patients after hip fractures [48]. Although Ali and Gibbons also found that ASA score being a predictor of readmission more robust than the CCI score or individual comorbidities [45]. However, we found the association with readmission was stronger with CCI score than with ASA score.

In the literature, large disparities exist regarding surgical complications or reoperation rates after hemiarthroplasty for unstable trochanteric fractures [9–11, 13–15, 18, 24, 27, 30]. The 6-month surgical complication/reoperation rates are ~ 2.2% [24]; one-year surgical complication/reoperation rates are from 2.6 to 20% [10, 13, 14] and 2-year surgical complication/reoperation rates are from 2.4 to 18.3% [9, 18, 27, 30]. Surgical site infection, dislocation and periprosthetic fracture are three major causes of the reoperations [9–11, 13–15, 18, 24, 27, 30]. The 1-year reoperation rates are from 2.9 to 16.3% after hemiarthroplasty for displaced femoral neck fracture in the literature [19, 49, 50]. By contrast, in our study, older age was a protective factor for reoperation. For each yearly increase in age, the sub-distribution hazard ratio (sHR) dropped by 1.4% (sHR: 0.986, 95% CI: 0.974–0.998) for reoperation. Again, the different directions of the risks between the long-term mortality and the reoperation were due to the competing risk of death. Fragile patients have higher risk for mortality. Therefore, in the risk set, if healthy patients formed a greater proportion, the overall chance of the set for operation became lower. The competing risk of death usually has a larger impact on the outcomes of long-term than of short-term. We did not identify other significant risk factors for reoperation except for age effect. Competing risk of death partly explains for that. With respect to surgical complication, hemiarthroplasty is likely more robust than internal fixation among the heterogeneous population. Therefore no particular subgroup showed higher risk for reoperation after hemiarthroplasty. Although hemiarthroplasty for unstable trochanteric fracture is relatively rare and the procedures are technically more challenging, our reoperation rates are still comparable to hemiarthroplasty after femoral neck fracture.

Therefore, compared with previous studies, the mortality and revision rates after hemiarthroplasty for unstable trochanteric fracture were comparable to treatment with internal fixation for trochanteric fracture or hemiarthroplasty for femoral neck fracture. Hemiarthroplasty might be a salvage procedure in difficult situations, such as poor bone quality or while a multi-fragmentary or comminuted fracture is occurred.

### Limitations

Our retrospective population study has some limitations. The standard of care for patients with (unstable) trochanteric fractures is usually intramedullary fixation. Unlike other case-control or cohort studies, we did not use the patients with trochanteric fracture receiving intramedullary fixation as controls. The main reason is that we cannot differentiate the stable or unstable trochanteric fractures and the severity of bone injury and comorbidities according to the ICD-9-CM codes. In our NIH program, all the hemiarthroplasty operations were required to be approved in advance by at least 3 orthopedic surgeons through peer-review system. In addition, no pre-approval was required for internal fixation. And no standard criteria or NHI guidance existed for the diagnosis and implant selection (intramedullary fixation or others) of unstable trochanteric fracture for internal fixation. Therefore, no valid control group can be selected based on our database. However, we can identify the fracture type of patients with trochanteric fracture receiving hemiarthroplasty as unstable trochanteric fracture from our database based on the NHI guidance. We provided a comparison of baseline characteristics of patients receiving hemiarthroplasty and internal fixation for trochanteric hip fractures in a supplemental table (Table S1) to differentiate the characteristics of this fragile unstable trochanteric patients receiving hemiarthroplasty from others. As shown in Table S1, there were more females, more patients with avascular necrosis of femoral head, and more patients with dementia among patients receiving hemiarthroplasty for trochanteric hip fractures. The reasons might because these patients may be due to more osteoporosis of bone quality, more comminuted fracture, pre-injury malfunction of hip joint, and inability to protection of injured hip by themselves. Besides, our study patients were followed up for various durations (2 years to 10 years), some unknown confounding factors might change during various follow-up periods. Lastly, the database does not contain some clinical parameters and other risk factors, such as body mass index, laboratory data, pre-operative activity, functional score, smoking, living environment, bone mineral density, severity of comorbidity, walking ability, quality of life, waiting time to surgery, duration time of surgery, blood loss, and blood transfusion. Therefore, unknown confounding factors were not captured nor adjusted in our study.

## Conclusions

This is the first study to assess the long-term effects of unstable trochanteric fractures treated with hemiarthroplasty. Male gender, older age and higher CCI score and lower insured amount were risk factors for the overall mortality. Older age and higher CCI score were risk factors for the first readmission. Conversely, we found that male gender was not a risk factor for readmission and older age was a protective factor for reoperation. Both findings are likely due to the competing death. Other than age, we did not identify other significant risk factors for reoperation. Compared with previous studies, the mortality and revision rates after hemiarthroplasty for unstable trochanteric fracture are acceptable as a salvage procedure for this fragile sub-population. More prospective studies and clinical trials are required for the long-term outcomes after primary hemiarthroplasty for unstable trochanteric fractures.

## Supplementary Information

**Additional file 1: Table S1.** Comparison of baseline characteristics of patients receiving hemiarthroplasty and internal fixation for trochanteric hip fractures based on the population-based NHIRD database.

## Data Availability

The website of the Taiwan National Health Insurance Research Database is found at http://www.mohw.gov.tw/.

## Abbreviations

NHIRD: National Health Insurance Research Database
NRDD: National Register of Deaths Database
MOHW: Ministry of Health and Welfare Health and Welfare
NHI: National Health Insurance
ICD-9-CM: International Classification of Diseases, 9th Revision, Clinical Modification
CCI: Charlson comorbidity index
HR: Hazard ratio
CI: Confidence interval
sHR: Sub-distribution hazard ratio
ASA: American Society of Anesthesiologists

## References

[1] Abrahamsen B, van Staa T, Ariely R, Olson M, Cooper C (2009). Excess mortality following hip fracture: a systematic epidemiological review. Osteoporos Int.

[2] Tsuboi M, Hasegawa Y, Suzuki S, Wingstrand H, Thorngren KG (2007). Mortality and mobility after hip fracture in Japan: a ten-year follow-up. J Bone Joint Surg Br.

[3] Wang CB, Lin CF, Liang WM, Cheng CF, Chang YJ, Wu HC (2013). Excess mortality after hip fracture among the elderly in Taiwan: a nationwide population-based cohort study. Bone..

[4] Kaplan K, Miyamoto R, Levine BR, Egol KA, Zuckerman JD (2008). Surgical management of hip fractures: an evidence-based review of the literature. II: intertrochanteric fractures. J Am Acad Orthop Surg..

[5] Quinn RH, Mooar PA, Murray JN, Pezold R, Sevarino KS (2017). Treatment of hip fractures in the elderly. J Am Acad Orthop Surg.

[6] Ahrengart L, Törnkvist H, Fornander P, Thorngren KG, Pasanen L, Wahlström P (2002). A randomized study of the compression hip screw and gamma nail in 426 fractures. Clin Orthop Relat Res.

[7] Grau L, Summers S, Massel DH, Rosas S, Ong A, Hernandez VH (2018). Operative trends in the treatment of hip fractures and the role of Arthroplasty. Geriatr Orthop Surg Rehabil.

[8] Rodop O, Kiral A, Kaplan H, Akmaz I (2002). Primary bipolar hemiprosthesis for unstable intertrochanteric fractures. Int Orthop.

[9] Kim SY, Kim YG, Hwang JK (2005). Cementless calcar-replacement hemiarthroplasty compared with intramedullary fixation of unstable intertrochanteric fractures. A prospective, randomized study. J Bone Joint Surg Am.

[10] Chan KC, Gill GS (2000). Cemented hemiarthroplasties for elderly patients with intertrochanteric fractures. Clin Orthop Relat Res.

[11] Tang P, Hu F, Shen J, Zhang L, Zhang L (2012). Proximal femoral nail antirotation versus hemiarthroplasty: a study for the treatment of intertrochanteric fractures. Injury..

[12] Cankaya D, Ozkurt B, Tabak AY (2013). Cemented calcar replacement versus cementless hemiarthroplasty for unstable intertrochanteric femur fractures in the elderly. Ulus Travma Acil Cerrahi Derg..

[13] Görmeli G, Korkmaz MF, Görmeli CA, Adanaş C, Karataş T, Şimşek SA (2015). Comparison of femur intertrochanteric fracture fixation with hemiarthroplasty and proximal femoral nail systems. Ulus Travma Acil Cerrahi Derg.

[14] Esen E, Dur H, Ataoğlu MB, Ayanoğlu T, Turanlı S (2017). Evaluation of proximal femoral nail-antirotation and cemented, bipolar hemiarthroplasty with calcar replacement in treatment of intertrochanteric femoral fractures in terms of mortality and morbidity ratios. Eklem Hastalik Cerrahisi.

[15] Kim JT, Kim HH, Kim JH, Kwak YH, Chang EC, Ha YC (2018). Mid-term survivals after cementless bipolar hemiarthroplasty for unstable intertrochanteric fractures in elderly patients. J Arthroplast.

[16] Cobden A, Camurcu Y, Duman S, Kocabiyik A, Kıs M, Saklavcı N (2019). Mid-term survivals of cemented calcar-replacement bipolar hemiarthroplasty for unstable intertrochanteric fractures in elderly patients. Injury..

[17] Camurcu Y, Cobden A, Sofu H, Saklavci N, Kis M (2017). What are the determinants of mortality after cemented bipolar Hemiarthroplasty for unstable intertrochanteric fractures in elderly patients?. J Arthroplast.

[18] Mansukhani SA, Tuteja SV, Kasodekar VB, Mukhi SR (2017). A comparative study of the dynamic hip screw, the cemented bipolar Hemiarthroplasty and the proximal femoral nail for the treatment of unstable intertrochanteric fractures. J Clin Diagn Res.

[19] Lin JC, Liang WM (2015). Outcomes after fixation for undisplaced femoral neck fracture compared to hemiarthroplasty for displaced femoral neck fracture among the elderly. BMC Musculoskelet Disord.

[20] Kulkarni GS, Limaye R, Kulkarni M, Kulkarni S (2006). Current concept review: intertrochanteric fractures. Indian J Orthop.

[21] Müller ME, Nazarian S, Koch P, Schatzker J (1990). The comprehensive classification of fractures of long bones.

[22] Beyersmann J, Allignol A, Schumacher M (2012). Competing risks and multistate models with R.

[23] Ranstam J, Kärrholm J, Pulkkinen P, Mäkelä K, Espehaug B, Pedersen AB, Mehnert F, Furnes O, for the NARA study group (2011). Statistical analysis of arthroplasty data. II Guidelines Acta Orthop.

[24] Bonnevialle P, Saragaglia D, Ehlinger M, Tonetti J, Maisse N, Adam P, le Gall C (2011). Trochanteric locking nail versus arthroplasty in unstable intertrochanteric fracture in patients aged over 75 years. Orthop Traumatol Surg Res.

[25] Desteli EE, İmren Y, Erdoğan M, Aydagün Ö (2015). Quality of life following treatment of trochanteric fractures with proximal femoral nail versus cementless bipolar hemiarthroplasty in elderly. Clin Invest Med.

[26] Park BJ, Cho HM, Min WB (2015). A comparison of internal fixation and bipolar Hemiarthroplasty for the treatment of reverse oblique intertrochanteric femoral fractures in elderly patients. Hip Pelvis.

[27] Lee YK, Ha YC, Chang BK, Kim KC, Kim TY, Koo KH (2011). Cementless bipolar hemiarthroplasty using a hydroxyapatite-coated long stem for osteoporotic unstable intertrochanteric fractures. J Arthroplast.

[28] Xie Y, Zhou H (2020). Primary cemented hemiarthroplasty for unstable intertrochanteric fractures in elderly severe osteoporotic patients. Injury..

[29] Fox KM, Magaziner J, Hebel JR, Kenzora JE, Kashner TM (1999). Intertrochanteric versus femoral neck hip fractures: differential characteristics, treatment, and sequelae. J Gerontol A Biol Sci Med Sci.

[30] Kim JW, Shon HC, Song SH, Lee YK, Koo KH, Ha YC (2020). Reoperation rate, mortality and ambulatory ability after internal fixation versus hemiarthroplasty for unstable intertrochanteric fractures in elderly patients: a study on Korean hip fracture registry. Arch Orthop Trauma Surg.

[31] Cornwall R, Gilbert MS, Koval KJ, Strauss E, Siu AL (2004). Functional outcomes and mortality vary among different types of hip fractures: a function of patient characteristics. Clin Orthop Relat Res.

[32] von Friesendorff M, Besjakov J, Akesson K (2008). Long-term survival and fracture risk after hip fracture: a 22-year follow-up in women. J Bone Miner Res.

[33] Forte ML, Virnig BA, Swiontkowski MF, Bhandari M, Feldman R, Eberly LE, Kane RL (2010). Ninety-day mortality after intertrochanteric hip fracture: does provider volume matter?. J Bone Joint Surg Am.

[34] Mundi S, Pindiprolu B, Simunovic N, Bhandari M (2014). Similar mortality rates in hip fracture patients over the past 31 years. Acta Orthop.

[35] Li AB, Zhang WJ, Wang J, Guo WJ, Wang XH, Zhao YM (2017). Intramedullary and extramedullary fixations for the treatment of unstable femoral intertrochanteric fractures: a meta-analysis of prospective randomized controlled trials. Int Orthop.

[36] Tucker A, Donnelly KJ, Rowan C, McDonald S, Foster AP (2018). Is the best plate a nail? A review of 3230 unstable intertrochanteric fractures of the proximal femur. J Orthop Trauma.

[37] Mattisson L, Bojan A, Enocson A (2018). Epidemiology, treatment and mortality of trochanteric and subtrochanteric hip fractures: data from the Swedish fracture register. BMC Musculoskelet Disord.

[38] Frisch NB, Wessell N, Charters M, Greenstein A, Shaw J, Peterson E, Trent Guthrie S (2018). Hip fracture mortality: differences between intertrochanteric and femoral neck fractures. J Surg Orthop Adv.

[39] Haentjens P, Magaziner J, Colón-Emeric CS, Vanderschueren D, Milisen K, Velkeniers B, Boonen S (2010). Meta-analysis: excess mortality after hip fracture among older women and men. Ann Intern Med.

[40] Radley DC, Gottlieb DJ, Fisher ES, Tosteson AN (2008). Comorbidity risk-adjustment strategies are comparable among persons with hip fracture. J Clin Epidemiol.

[41] Kirkland LL, Kashiwagi DT, Burton MC, Cha S, Varkey P (2011). The charlson comorbidity index score as a predictor of 30-day mortality after hip fracture surgery. Am J Med Qual.

[42] Cram P, Lix LM, Bohm E, Yan L, Roos L, Matelski J, Gandhi R, Landon B, Leslie WD (2019). Hip fracture care in Manitoba, Canada and New York state, United States: an analysis of administrative data. CMAJ Open.

[43] Kristensen MT, Öztürk B, Röck ND, Ingeman A, Palm H, Pedersen AB (2019). Regaining pre-fracture basic mobility status after hip fracture and association with post-discharge mortality and readmission-a nationwide register study in Denmark. Age Ageing.

[44] Bohl DD, Basques BA, Golinvaux NS, Miller CP, Baumgaertner MR, Grauer JN (2014). Extramedullary compared with intramedullary implants for intertrochanteric hip fractures: thirty-day outcomes of 4432 procedures from the ACS NSQIP database. J Bone Joint Surg Am.

[45] Ali AM, Gibbons CE (2017). Predictors of 30-day hospital readmission after hip fracture: a systematic review. Injury..

[46] Pollock FH, Bethea A, Samanta D, Modak A, Maurer JP, Chumbe JT (2015). Readmission within 30 days of discharge after hip fracture care. Orthopedics..

[47] Lizaur-Utrilla A, Gonzalez-Navarro B, Vizcaya-Moreno MF, Miralles Muñoz FA, Gonzalez-Parreño S, Lopez-Prats FA (2019). Reasons for delaying surgery following hip fractures and its impact on one year mortality. Int Orthop.

[48] French DD, Bass E, Bradham DD, Campbell RR, Rubenstein LZ (2008). Rehospitalization after hip fracture: predictors and prognosis from a national veterans study. J Am Geriatr Soc.

[49] Gao H, Liu Z, Xing D, Gong M (2012). Which is the best alternative for displaced femoral neck fractures in the elderly?: a meta-analysis. Clin Orthop Relat Res.

[50] Bhandari M, Einhorn TA, Guyatt G, Schemitsch EH, Zura RD, Sprague S (2019). Total hip Arthroplasty or Hemiarthroplasty for hip fracture. N Engl J Med.

