# Supplementary material for: Primary hemiarthroplasty after unstable trochanteric fracture in elderly patients: mortality, readmission and reoperation
Source: BMC Musculoskelet Disord. 2021 Apr 30;22:403. doi: 10.1186/s12891-021-04277-7 (PMC8091504; doi:10.1186/s12891-021-04277-7)
Supplement: Supplementary file 1 — Additional file 1: Table S1. Comparison of baseline characteristics of patients receiving hemiarthroplasty and internal fixation for trochanteric hip fractures based on the population-based NHIRD database. [file 12891_2021_4277_MOESM1_ESM.docx]

**Table S1** Comparison of baseline characteristics of patients receiving hemiarthroplasty and internal fixation for trochanteric hip fractures based on the population-based NHIRD database

|  |  | Trochanteric(n=76,798) | |  |
| --- | --- | --- | --- | --- |
|  |  | Hemiarthroplasty | Internal Fixation |  |
|  |  | (n=2,798) | (n=74,000) |  |
|  |  | N(%) | N(%) | *P*-value |
| Age (yrs) | 60-64 | 113(4.04) | 3272(4.42) | 0.066 |
|  | 65-69 | 226(8.08) | 6065(8.2) |  |
|  | 70-74 | 407(14.55) | 11377(15.37) |  |
|  | 75-79 | 599(21.41) | 16963(22.92) |  |
|  | 80-84 | 737(26.34) | 17938(24.24) |  |
|  | ≥ 85 | 716(25.59) | 18385(24.84) |  |
| Gender | Male | 895(31.99) | 31720(42.86) | <0.001 |
|  | Female | 1903(68.01) | 42280(57.14) |  |
| CCI^b^ score | 0 | 917(32.77) | 24608(33.25) | 0.180 |
|  | 1 | 694(24.8) | 17795(24.05) |  |
|  | 2 | 372(13.3) | 10843(14.65) |  |
|  | 3 | 228(10.29) | 7670(10.36) |  |
|  | ≥4 | 527(18.83) | 13084(17.68) |  |
| Arthropathy | Osteoarthritis | 288(10.29) | 7156(9.67) | 0.274 |
|  | Inflammatory arthritis^c^ | 26(0.93) | 742(1.00) | 0.701 |
|  | AVNF^d^ | 19(0.68) | 237(0.32) | 0.001 |
| Comorbidities affecting mobility^e^ | Cerebrovascular disease | 681(24.34) | 17363(23.46) | 0.284 |
|  | Dementia | 186(6.65) | 3928(5.31) | 0.002 |
|  | Hemiplegia | 154(5.5) | 3741(5.06) | 0.289 |
|  | Moderate to severe renal disease | 243(8.68) | 5958(8.05) | 0.227 |

*Note*: ^a^ SD: standard deviation. ^b^ CCI: Charlson Comorbidity Index.

^c^ Inflammatory arthritis (rheumatoid arthritis, systemic lupus erythematosus, ankylosing arthritis) ^d^ Avascular necrosis of femoral head.

^e^ pre-injury residential status

Data Source: National Health Insurance Research Database (NHIRD) (details available at: http://nhird.nhri.org.tw/en/index.htm). All patients’ claims were collected within the time frame of 2000 to 2010.
